# Crystal structure of 2-oxo-2*H*-chromen-7-yl 4-fluoro­benzoate

**DOI:** 10.1107/S205698901800614X

**Published:** 2018-04-27

**Authors:** Akoun Abou, Jules Yoda, Abdoulaye Djandé, Stéphane Coussan, T. Jérémie Zoueu

**Affiliations:** a Unité Mixte de Recherche et d’Innovation en Electronique et d’Electricité Appliqueés (UMRI EEA), Equipe de Recherche: Instrumentation Image et Spectroscopie (L2IS), DFR–GEE, Institut National Polytechnique Félix Houphouët-Boigny (INPHB), BP 1093 Yamoussoukro, Côte d’Ivoire; b Laboratoire de Chimie Moléculaire et de Matériaux (LCMM), Equipe de Chimie Organique et de Phytochimie, Université Ouaga I Pr Joseph KI-ZERBO, 03 BP 7021 Ouagadougou 03, Burkina Faso; c CNRS, Aix-Marseille Université, UMR 7345, Laboratoire de Physique des Interactions Ioniques et Moléculaires, Centre St Jérôme, 13397 Marseille Cedex 20, France

**Keywords:** coumarin ester, C—H⋯O hydrogen bonds, π–π stacking inter­actions, Hirshfeld surface analysis, quantum chemical calculations, crystal structure

## Abstract

In the title compound, C_16_H_9_FO_4_, (I), the benzene ring is oriented at an acute angle of 59.03 (15)° relative to the coumarin plane (r.m.s deviation = 0.009 Å). This conformation of (I) is stabilized by an intra­molecular C—H⋯O hydrogen bond, which closes a five-membering ring. In the crystal, mol­ecules of (I) form infinite zigzag chains along the *b-*axis direction, linked by C—H⋯O hydrogen bonds. Furthermore, the crystal structure is supported by π–π stacking inter­actions between neighbouring pyrone and benzene or coumarin rings [centroid–centroid distances in the range 3.5758 (18)–3.6115 (16) Å], as well as C=O⋯π inter­actions [O⋯centroid distances in the range 3.266 (3)–3.567 (3) Å]. The theoretical data for (I) obtained from quantum chemical calculations are in good agreement with the observed structure, although the calculated C—O—C—C torsion angle between the coumarin fragment and the benzene ring (73.7°) is somewhat larger than the experimental value [63.4 (4)°]. Hirshfeld surface analysis has been used to confirm and qu­antify the supra­molecular inter­actions.

## Abstract 2

The structure of a coumarin ester stabilized by C—H⋯O hydrogen bonds and C=O⋯π and π–π stacking inter­actions has been studied by X-ray diffraction, Hirshfeld surface analysis and quantum chemical calculations.

## Chemical context   

Coumarins and their derivatives constitute one of the major classes of naturally occurring compounds and inter­est in their chemistry continues unabated because of their usefulness as biologically active agents. They also form the core of several mol­ecules of pharmaceutical importance. Coumarin and its derivatives have been reported to serve as anti-bacterial (Basanagouda *et al.*, 2009 ▸), anti-oxidant (Vuković *et al.*, 2010 ▸) and anti-inflammatory agents (Emmanuel-Giota *et al.*, 2001 ▸). In view of their importance and as a continuation of our work on the crystal structure analysis of coumarin derivatives (Abou *et al.*, 2013 ▸; Ouédraogo *et al.*, 2018 ▸), we report herein the synthesis, crystal structure, geometry optimization and Hirshfeld surface analysis of the title coumarin derivative (I).

## Structural commentary   

**Figure 1:**
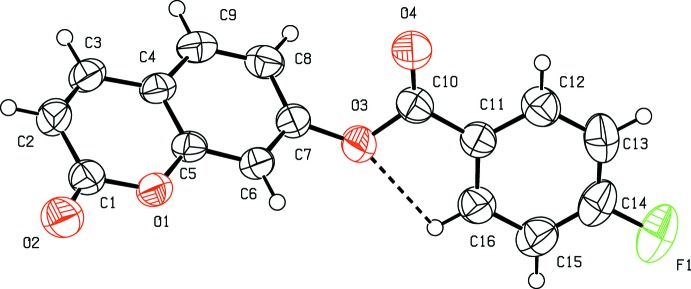
The mol­ecular structure of (I), along with the atomic numbering scheme. Displacement ellipsoids are drawn at the 50% probability level. H atoms are shown as spheres of arbitrary radius. The intra­molecular hydrogen bond is indicated by a dashed line.

The mol­ecular structure of (I) is illustrated in Fig. 1 ▸. In the structure, an *S*(5) ring motif arises from the intra­molecular C16—H16⋯O3 hydrogen bond (Table 1 ▸), and generates a pseudo bicyclic ring system (Fig. 1 ▸). The coumarin fragment is planar (r.m.s deviation = 0.009 Å) and oriented at an acute angle of 59.03 (15)° with respect to the C11–C16 benzene ring, while the hydrogen-bonded five-membered ring [r.m.s deviation = 0.007 Å] forms dihedral angles of 59.23 (13) and 0.59 (18)°, respectively, with the coumarin ring system and the benzene ring. These dihedral angles suggest that the five-membered hydrogen-bonded and C11–C16 benzene rings are coplanar. An inspection of the bond lengths shows that there is a slight asymmetry of the electronic distribution around the pyrone ring: the C2—C3 [1.332 (5) Å] and C1—C2 [1.451 (5) Å] bond lengths are shorter and longer, respectively, than those expected for a C_ar_—C_ar_ bond. This suggests that the electron density is preferentially located in the C3—C2 bond of the pyrone ring, as seen in other coumarin derivatives (Gomes *et al.*, 2016 ▸; Ziki *et al.*, 2016 ▸).
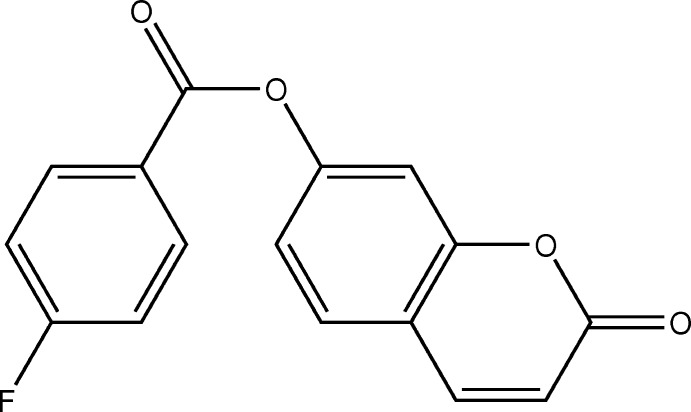


## Supra­molecular features   

**Figure 2:**
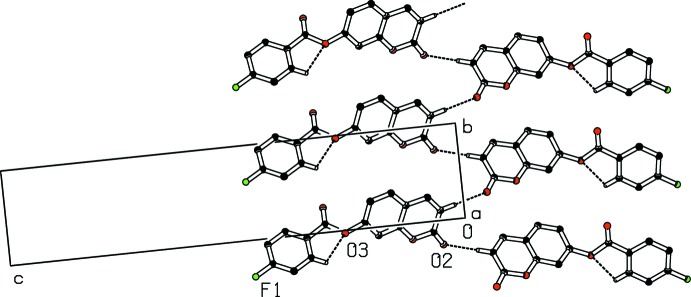
Part of the crystal packing of (I) showing the formation of an infinite *C*(4) chain along the *b*-axis. Dashed lines indicate hydrogen bonds. H atoms not involved in hydrogen-bonding inter­actions have been omitted for clarity.

**Figure 3:**
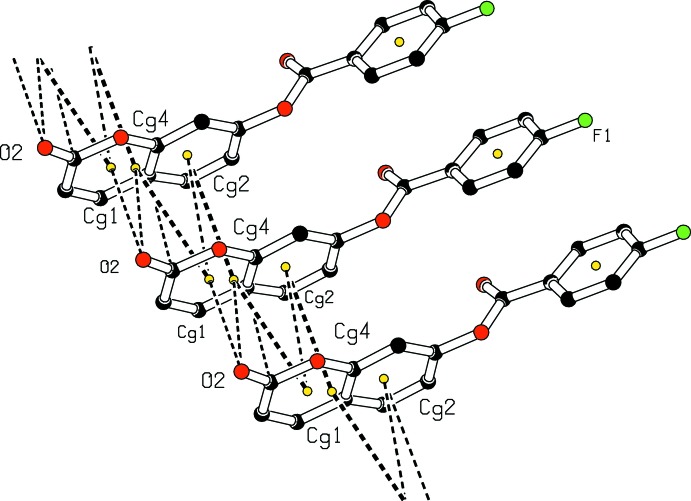
A view of the crystal packing showing C1=O2⋯π and π–π stacking inter­actions (dashed lines). The yellow dots are ring centroids.

In the crystal, the C2—H2⋯O2 hydrogen bond links mol­ecules into infinite zigzag *C*(4) chains along the [010] direction (Fig. 2 ▸). In addition, a close contact with a distance shorter than the sum of the van der Waals radii [C1⋯C4 (−1 + *x*, *y*, *z*) = 3.336 (5) Å] and C1=O2⋯π inter­actions are present [O2⋯*Cg*1 (−1 + *x*, *y*, *z*) = 3.266 (3) and O2⋯*Cg*4 (−1 + *x*, *y*, *z*) = 3.567 (3) Å, where *Cg*1 and *Cg*4 are the centroids of the pyrone ring and the coumarin ring system, respectively]. The resulting supra­molecular aggregation is completed by the presence of π–π stacking between the pyrone and C4–C9 benzene rings or coumarin ring systems (Fig. 3 ▸). The centroid–centroid distances [*Cg*1⋯*Cg*2 (−1 + *x*, *y*, *z*) = 3.5758 (18), *Cg*1⋯*Cg*4 (−1 + *x*, *y*, *z*) = 3.6116 (16), *Cg*2⋯*Cg*4 (1 + *x*, *y*, *z*) = 3.6047 (16) Å, where *Cg*2 is the centroid of the C4–C9 benzene ring] are less than 3.8 Å, the maximum regarded as suitable for an effective π–π inter­action (Janiak, 2000 ▸). The perpendicular distances of *Cg*(*I*) on ring *J* and distances between *Cg*(*I*) and perpendicular projection of *Cg*(*J*) on ring *I* (slippage) are summarized in Table 2 ▸.

## Database survey   

A CSD search (Web CSD version 5.39; March 9, 2018; Groom *et al.*, 2016 ▸) found five coumarin ester structures with substituents at the 7 position (Ramasubbu *et al.*, 1982 ▸; Gnanaguru *et al.*, 1985 ▸; Parveen *et al.*, 2011 ▸; Ji *et al.*, 2014 ▸, 2017 ▸). In these structures and those of *meta*-substituted coumarin esters (Abou *et al.*, 2013 ▸; Bibila Mayaya Bisseyou *et al.*, 2013 ▸; Yu *et al.*, 2014 ▸; Gomes *et al.*, 2016 ▸; Ziki *et al.*, 2016 ▸, 2017 ▸), the pyrone rings show three long (in the range 1.37–1.46 Å) and one short (1.32–1.34 Å) C—C distances, suggesting that the electronic density is preferentially located in the short C—C bond at the pyrone ring. This pattern is clearly repeated for (I) with C2—C3 = 1.332 (5) Å, while C1—C2 = 1.451 (5), C3—C4 = 1.434 (4) and C4—C5 = 1.399 (4) Å.

## Hirshfeld surface analysis   

**Figure 4:**
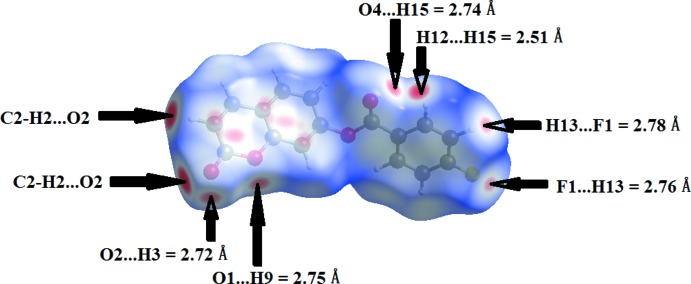
A view of the Hirshfeld surface for (I) with the three-dimensional *d*_norm_ surfaces mapped over a fixed colour scale of −0.26 (red) to 1.20 Å (blue).

**Figure 5:**
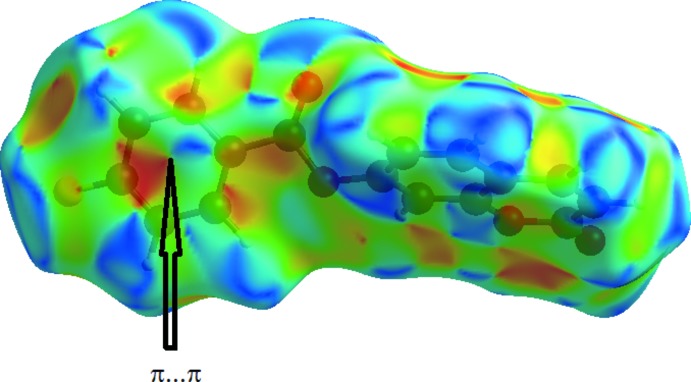
Hirshfeld surface mapped over shape-index highlighting the regions involved in π–π stacking inter­actions.

**Figure 6:**
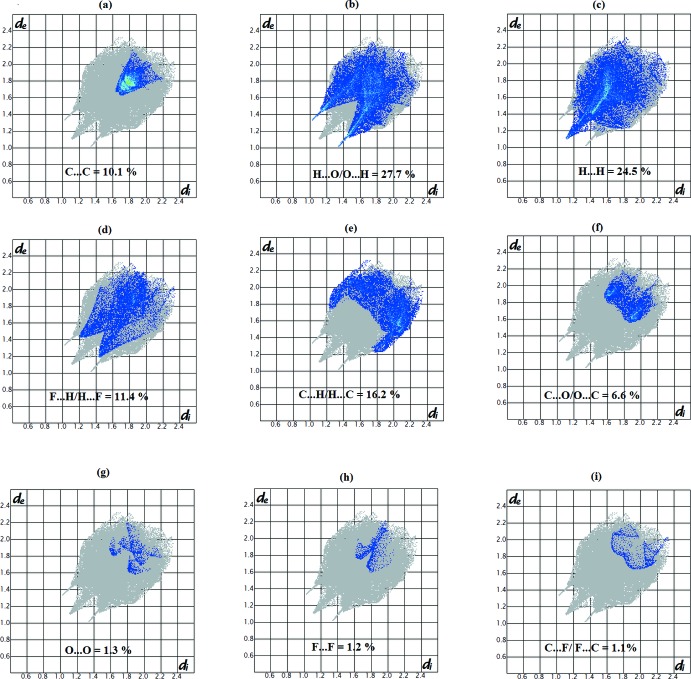
Decomposed two-dimensional fingerprint plots for (I). Various short contacts and their relative contributions are indicated.

Mol­ecular Hirshfeld surfaces and the associated two-dimensional fingerprint plots of (I) were calculated using a standard (high) surface resolution with the the three-dimensional *d*_norm_ surfaces mapped over a fixed colour scale of −0.26 (red) to 1.20 Å (blue) with the program *CrystalExplorer 3.1* (Wolff *et al.*, 2012 ▸). The analysis of inter­molecular inter­actions through the mapping of three-dimensional *d*_norm_ surfaces is permitted by the contact distances *d*_i_ and *d*_e_ from the Hirshfeld surface to the nearest atom inside and outside, respectively. In (I), the surface mapped over *d*_norm_ highlights several red spots showing distances shorter than the sum of the van der Waals radii. These dominant inter­actions correspond to inter­molecular C—H⋯O hydrogen bonds, C8⋯C5 (1 + *x*, *y*, *z*), O⋯π and π–π stacking inter­actions between the surface and the neighbouring environment. The mapping also shows white or pale-red spots with distances almost equal to the sum of the van der Waals radii and blue regions with distances longer than the sum of the van der Waals radii. The surfaces are shown as transparent to allow visualization of the mol­ecule (Fig. 4 ▸). In the shape-index map (−0.99 to 1 Å) (Fig. 5 ▸), the adjacent red and blue triangle-like patches show concave regions that indicate π–π stacking inter­actions (Bitzer *et al.*, 2017 ▸). Furthermore, the 2D fingerprint plots (FP), decomposed to highlight particular close contacts of atom pairs and the contributions from different contacts, are provided in Fig. 6 ▸. The red spots in the middle of the surface appearing near *d*_e_ = *d*_i_ = 1.8-2.0 Å correspond to close C⋯C inter­planar contacts. These contacts, which comprise 10.1% of the total Hirshfeld surface area, are related to π–π inter­actions (Fig. 6 ▸ *a*) as predicted by the X-ray study. The most significant contrib­ution to the Hirshfeld surface (27.7%) is from H⋯O/O⋯H contacts, which appear on the left-side as blue spikes with the tip at *d*_e_ + *d*_i_ = 2.4 Å, top and bottom (Fig. 6 ▸ *b*). As expected in organic compounds, the H⋯H contacts are important with a 24.5% contribution to Hirshfeld surface; these appear in the central region of the FP with a central blue tip spike at *d*_e_ = *d*_i_ = 1.10 Å (Fig. 6 ▸ *c*) whereas the F⋯H/H⋯F contacts with a contribution to the Hirshfeld surface of 11.4% are indicated by the distribution of points around a pair of wings at *d*_e_ + *d*_i_ 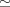 2.6 Å (Fig. 6 ▸ *d*). The C⋯H/H⋯C plot (16.2%) reveals information on the inter­molecular hydrogen bonds (Fig. 6 ▸ *e*). Other visible spots in the Hirshfeld surfaces indicate the C⋯O/O⋯C, O⋯O, F⋯F and C⋯F/F⋯C contacts, which contribute only 6.6, 1.3, 1.2 and 1.1%, respectively (Fig. 6 ▸ *f*–6*i*).

## Theoretical calculations   

The geometry optimization of (I) was performed using the density functional theory (DFT) method with a 6-311^++^G(d,p) basis set. The crystal structure in the solid state was used as the starting structure for the calculations. The DFT calculations were performed with the *GAUSSIAN09* program package (Frisch *et al.*, 2013 ▸). The resulting geometrical parameters are compared with those obtained from the X-ray crystallographic study, showing a good agreement for the bond lengths and bond angles with r.m.s. deviations of 0.017 Å and 1.06°, respectively (see Supplementary Tables S1 and S2). In addition, an inspection of the calculated torsion angles shows that the coumarin fragment and the C11–C16 benzene ring are co-planar (see Supplementary Table S3), which is in good agreement with the experimental results, although the calculated C10—O3—C7—C8 torsion angle between them (73.7°) is somewhat larger than the observed value [63.4 (4)°].

## Synthesis and crystallization   

To a solution of 4-fluoro­benzoyl chloride (6.17 mmol; 0.98 g) in dried tetra­hydro­furan (40 mL) was added dried tri­ethyl­amine (3 molar equivalents; 2.6 mL) and 7-hy­droxy­coumarin (6.17 mmol; 1 g) by small portions over 30 min. The mixture was then refluxed for 4 h and poured into 40 mL of chloro­form. The solution was acidified with diluted hydro­chloric acid until the pH was 2–3. The organic layer was extracted, washed with water to neutrality, dried over MgSO_4_. The resulting precipitate (crude product) was filtered off with suction, washed with petroleum ether and recrystallized from acetone. Pale-yellow crystals of (I) were obtained in a good yield (85.1%; m.p. 467–468 K).

## Refinement details   

Crystal data, data collection and structure refinement details are summarized in Table 3 ▸. H atoms were placed in calculated positions (C—H = 0.93 Å) and refined using the riding-model approximation with *U*_iso_(H) = 1.2*U*_eq_(C).

## Supplementary Material

Crystal structure: contains datablock(s) I. DOI: 10.1107/S205698901800614X/kq2021sup1.cif

Structure factors: contains datablock(s) I. DOI: 10.1107/S205698901800614X/kq2021Isup2.hkl

Supporting information file. DOI: 10.1107/S205698901800614X/kq2021Isup3.cml

CCDC reference: 1834035

Additional supporting information:  crystallographic information; 3D view; checkCIF report

## Figures and Tables

**Table 1:** Hydrogen-bond geometry (Å, °) *Cg*2 and *Cg*4 are the centroids of the C4–C9 benzene ring and the coumarin ring system, respectively.

*D*—H⋯*A*	*D*—H	H⋯*A*	*D*⋯*A*	*D*—H⋯*A*
C16—H16⋯O3	0.93	2.37	2.693 (4)	100
C2—H2⋯O2^i^	0.93	2.51	3.412 (4)	163
C1—O2⋯*Cg*2^ii^	1.20 (1)	3.27 (1)	3.403 (3)	86 (1)
C1—O2⋯*Cg*4^ii^	1.20 (1)	3.57 (1)	3.368 (3)	71 (1)

Symmetry codes: (i) 
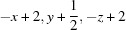
; (ii) 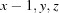.

**Table 2:** Analysis of short ring inter­actions (Å)

*Cg*(*I*)	*Cg*(*J*)	Symmetry *Cg*(*J*)	*Cg*(*I*)⋯*Cg*(*J*)	*CgI*_Perp	*CgJ*_Perp	Slippage
*Cg*1	*Cg*2	−1 + *x*, *y*, *z*	3.5758 (18)	3.3139 (13)	−3.3124 (13)	1.347
*Cg*1	*Cg*4	−1 + *x*, *y*, *z*	3.6116 (16)	3.3133 (13)	−3.3044 (10)	1.458
*Cg*2	*Cg*1	1 + *x*, *y*, *z*	3.5758 (18)	−3.3123 (13)	3.3140 (13)	1.343
*Cg*2	*Cg*4	1 + *x*, *y*, *z*	3.6047 (16)	−3.3109 (13)	3.3195 (10)	1.405
*Cg*4	*Cg*1	1 + *x*, *y*, *z*	3.6115 (16)	−3.3043 (10)	3.3134(13	1.437
*Cg*4	*Cg*2	−1 + *x*, *y*, *z*	3.6049 (16)	3.3196 (10)	−3.3110 (13)	1.426

*Cg*(*I*) and *Cg*(*J*) are centroids of rings *I* and *J*; *CgI*_Perp is the perpendicular distance of *Cg*(*I*) on ring *J* and slippage is the distance between *Cg*(*I*) and the perpendicular projection of *Cg*(*J*) on ring *I*.

**Table 3:** Experimental details

Crystal data	
Chemical formula	C_16_H_9_FO_4_
*M*_r_	284.23
Crystal system, space group	Monoclinic, *P*2_1_
Temperature (K)	298
*a*, *b*, *c* (Å)	4.0181 (2), 5.7296 (3), 27.5566 (14)
β (°)	91.660 (4)
*V* (Å^3^)	634.14 (6)
*Z*	2
Radiation type	Cu *K*α
μ (mm^−1^)	1.00
Crystal size (mm)	0.40 × 0.12 × 0.05

Data collection	
Diffractometer	Rigaku SuperNova, Dual, Cu at zero, Atlas S2
Absorption correction	Multi-scan (*CrysAlis PRO*; Rigaku OD, 2015 ▸)
*T*_min_, *T*_max_	0.683, 1.000
No. of measured, independent and observed [*I* > 2σ(*I*)] reflections	8239, 2228, 2149
*R*_int_	0.026
(sin θ/λ)_max_ (Å^−1^)	0.601

Refinement	
*R*[*F* ^2^ > 2σ(*F* ^2^)], *wR*(*F* ^2^), *S*	0.035, 0.098, 1.13
No. of reflections	2228
No. of parameters	190
No. of restraints	1
H-atom treatment	H-atom parameters constrained
Δρ_max_, Δρ_min_ (e Å^−3^)	0.13, −0.16
Absolute structure	Flack *x* determined using 875 quotients [(*I* ^+^)−(*I* ^−^)]/[(*I* ^+^)+(*I* ^−^)] (Parsons *et al.*, 2013 ▸)
Absolute structure parameter	−0.03 (8)

Computer programs: *CrysAlis PRO* (Rigaku OD, 2015 ▸), *SIR2014* (Burla *et al.*, 2015 ▸), *SHELXL2014* (Sheldrick, 2015 ▸), *PLATON* (Spek, 2009 ▸), *Mercury* (Macrae *et al.*, 2008 ▸), *publCIF* (Westrip, 2010 ▸) and *WinGX* (Farrugia, 2012 ▸).

## supplementary crystallographic information

### Crystal data

**Table:**

C_16_H_9_FO_4_	*F*(000) = 292
*M**_r_* = 284.23	*D*_x_ = 1.489 Mg m^−^^3^
Monoclinic, *P*2_1_	Melting point = 467–468 K
Hall symbol: P2yb	Cu *K*α radiation, λ = 1.54184 Å
*a* = 4.0181 (2) Å	Cell parameters from 4751 reflections
*b* = 5.7296 (3) Å	θ = 4.8–67.5°
*c* = 27.5566 (14) Å	µ = 1.00 mm^−^^1^
β = 91.660 (4)°	*T* = 298 K
*V* = 634.14 (6) Å^3^	Prism, pale yellow
*Z* = 2	0.40 × 0.12 × 0.05 mm

### Data collection

**Table:**

Rigaku SuperNova, Dual, Cu at zero, Atlas S2 diffractometer	2228 independent reflections
Radiation source: micro-focus sealed X-ray tube	2149 reflections with *I* > 2σ(*I*)
Mirror monochromator	*R*_int_ = 0.026
Detector resolution: 5.3048 pixels mm^-1^	θ_max_ = 67.9°, θ_min_ = 4.8°
ω scans	*h* = −4→4
Absorption correction: multi-scan (CrysAlis PRO; Rigaku OD, 2015)	*k* = −6→6
*T*_min_ = 0.683, *T*_max_ = 1.000	*l* = −32→32
8239 measured reflections	

### Refinement

**Table:**

Refinement on *F*^2^	Secondary atom site location: difference Fourier map
Least-squares matrix: full	Hydrogen site location: inferred from neighbouring sites
*R*[*F*^2^ > 2σ(*F*^2^)] = 0.035	H-atom parameters constrained
*wR*(*F*^2^) = 0.098	*w* = 1/[σ^2^(*F*_o_^2^) + (0.0396*P*)^2^ + 0.1688*P*] where *P* = (*F*_o_^2^ + 2*F*_c_^2^)/3
*S* = 1.13	(Δ/σ)_max_ < 0.001
2228 reflections	Δρ_max_ = 0.13 e Å^−^^3^
190 parameters	Δρ_min_ = −0.16 e Å^−^^3^
1 restraint	Absolute structure: Flack *x* determined using 875 quotients [(*I*^+^)-(*I*^-^)]/[(*I*^+^)+(*I*^-^)] (Parsons *et al.*, 2013)
36 constraints	Absolute structure parameter: −0.03 (8)
Primary atom site location: structure-invariant direct methods	

### Special details

**Table:**

Geometry. All esds (except the esd in the dihedral angle between two l.s. planes) are estimated using the full covariance matrix. The cell esds are taken into account individually in the estimation of esds in distances, angles and torsion angles; correlations between esds in cell parameters are only used when they are defined by crystal symmetry. An approximate (isotropic) treatment of cell esds is used for estimating esds involving l.s. planes.

### Fractional atomic coordinates and isotropic or equivalent isotropic displacement parameters (Å^2^)

**Table:**

	*x*	*y*	*z*	*U*_iso_*/*U*_eq_	
O1	0.6427 (6)	0.3343 (4)	0.88096 (8)	0.0524 (6)	
O3	0.0412 (6)	0.4724 (4)	0.73539 (8)	0.0615 (6)	
C7	0.1548 (8)	0.5614 (6)	0.78012 (11)	0.0497 (7)	
C10	0.1085 (8)	0.5845 (6)	0.69372 (12)	0.0530 (8)	
C5	0.4431 (7)	0.4840 (5)	0.85424 (11)	0.0441 (6)	
O2	0.9356 (7)	0.2473 (5)	0.94698 (9)	0.0754 (8)	
C6	0.3531 (8)	0.4146 (6)	0.80778 (11)	0.0478 (7)	
H6	0.4246	0.2726	0.7955	0.057*	
C9	0.1406 (8)	0.8388 (6)	0.84388 (12)	0.0521 (7)	
H9	0.0667	0.9806	0.8560	0.062*	
C4	0.3401 (8)	0.6973 (5)	0.87346 (11)	0.0460 (7)	
C11	−0.0314 (8)	0.4585 (6)	0.65121 (11)	0.0503 (7)	
C16	−0.1991 (9)	0.2483 (7)	0.65592 (12)	0.0573 (8)	
H16	−0.2289	0.1853	0.6866	0.069*	
F1	−0.3952 (7)	0.1157 (6)	0.53091 (9)	0.1069 (10)	
O4	0.2703 (7)	0.7609 (5)	0.69274 (9)	0.0745 (8)	
C3	0.4468 (8)	0.7532 (6)	0.92219 (11)	0.0535 (8)	
H3	0.3784	0.8923	0.9361	0.064*	
C12	0.0095 (8)	0.5513 (7)	0.60524 (13)	0.0613 (9)	
H12	0.1210	0.6923	0.6018	0.074*	
C1	0.7538 (8)	0.3880 (6)	0.92768 (12)	0.0536 (8)	
C8	0.0489 (8)	0.7757 (6)	0.79730 (12)	0.0546 (8)	
H8	−0.0810	0.8743	0.7778	0.066*	
C14	−0.2763 (10)	0.2274 (9)	0.57115 (14)	0.0705 (11)	
C2	0.6429 (9)	0.6080 (7)	0.94771 (12)	0.0570 (8)	
H2	0.7103	0.6486	0.9792	0.068*	
C15	−0.3218 (10)	0.1325 (7)	0.61538 (14)	0.0679 (10)	
H15	−0.4339	−0.0085	0.6184	0.081*	
C13	−0.1144 (10)	0.4358 (9)	0.56453 (13)	0.0735 (11)	
H13	−0.0889	0.4972	0.5336	0.088*	

### Atomic displacement parameters (Å^2^)

**Table:**

	*U*^11^	*U*^22^	*U*^33^	*U*^12^	*U*^13^	*U*^23^
O1	0.0609 (13)	0.0430 (13)	0.0528 (12)	0.0028 (10)	−0.0050 (10)	−0.0037 (10)
O3	0.0801 (15)	0.0560 (15)	0.0479 (12)	−0.0166 (13)	−0.0091 (10)	0.0029 (11)
C7	0.0542 (16)	0.0476 (19)	0.0470 (16)	−0.0114 (15)	−0.0026 (13)	0.0003 (14)
C10	0.0528 (17)	0.052 (2)	0.0542 (18)	0.0026 (16)	−0.0010 (13)	0.0049 (15)
C5	0.0439 (14)	0.0383 (16)	0.0501 (15)	−0.0058 (12)	0.0007 (11)	0.0008 (12)
O2	0.0877 (19)	0.0643 (18)	0.0726 (16)	0.0051 (16)	−0.0235 (14)	0.0052 (14)
C6	0.0553 (16)	0.0396 (17)	0.0486 (16)	−0.0033 (13)	0.0039 (12)	−0.0033 (13)
C9	0.0527 (17)	0.0378 (17)	0.0660 (19)	0.0015 (13)	0.0063 (14)	−0.0016 (14)
C4	0.0484 (16)	0.0366 (17)	0.0532 (16)	−0.0052 (13)	0.0058 (12)	−0.0037 (12)
C11	0.0503 (16)	0.0493 (19)	0.0511 (16)	0.0079 (14)	−0.0034 (12)	0.0015 (14)
C16	0.0611 (19)	0.054 (2)	0.0567 (18)	0.0012 (17)	−0.0044 (14)	0.0021 (16)
F1	0.116 (2)	0.129 (3)	0.0749 (15)	−0.0067 (19)	−0.0203 (14)	−0.0386 (16)
O4	0.097 (2)	0.0636 (17)	0.0629 (15)	−0.0291 (16)	0.0016 (13)	0.0011 (13)
C3	0.0615 (19)	0.0438 (18)	0.0556 (18)	−0.0076 (16)	0.0066 (14)	−0.0096 (15)
C12	0.0592 (19)	0.064 (2)	0.060 (2)	−0.0040 (18)	−0.0001 (15)	0.0055 (17)
C1	0.0577 (18)	0.050 (2)	0.0527 (17)	−0.0078 (16)	−0.0050 (14)	0.0021 (15)
C8	0.0580 (19)	0.0442 (18)	0.0613 (19)	0.0014 (15)	−0.0029 (14)	0.0062 (14)
C14	0.067 (2)	0.081 (3)	0.063 (2)	0.004 (2)	−0.0124 (17)	−0.022 (2)
C2	0.065 (2)	0.056 (2)	0.0498 (17)	−0.0123 (16)	−0.0016 (14)	−0.0059 (15)
C15	0.070 (2)	0.061 (2)	0.072 (2)	−0.0016 (19)	−0.0067 (17)	−0.0090 (18)
C13	0.077 (2)	0.095 (3)	0.0480 (18)	0.011 (2)	−0.0041 (16)	0.002 (2)

### Geometric parameters (Å, º)

**Table:**

O1—C5	1.374 (3)	C11—C16	1.388 (5)
O1—C1	1.385 (4)	C11—C12	1.388 (5)
O3—C10	1.350 (4)	C16—C15	1.378 (5)
O3—C7	1.398 (4)	C16—H16	0.9300
C7—C6	1.374 (4)	F1—C14	1.355 (4)
C7—C8	1.387 (5)	C3—C2	1.332 (5)
C10—O4	1.202 (4)	C3—H3	0.9300
C10—C11	1.473 (4)	C12—C13	1.383 (5)
C5—C6	1.379 (4)	C12—H12	0.9300
C5—C4	1.399 (4)	C1—C2	1.451 (5)
O2—C1	1.202 (4)	C8—H8	0.9300
C6—H6	0.9300	C14—C15	1.352 (6)
C9—C8	1.373 (5)	C14—C13	1.374 (7)
C9—C4	1.388 (4)	C2—H2	0.9300
C9—H9	0.9300	C15—H15	0.9300
C4—C3	1.434 (4)	C13—H13	0.9300
			
C5—O1—C1	121.8 (2)	C11—C16—H16	119.8
C10—O3—C7	120.5 (3)	C2—C3—C4	120.7 (3)
C6—C7—C8	122.1 (3)	C2—C3—H3	119.6
C6—C7—O3	115.8 (3)	C4—C3—H3	119.6
C8—C7—O3	121.9 (3)	C13—C12—C11	120.5 (4)
O4—C10—O3	122.7 (3)	C13—C12—H12	119.7
O4—C10—C11	126.0 (3)	C11—C12—H12	119.7
O3—C10—C11	111.2 (3)	O2—C1—O1	116.0 (3)
O1—C5—C6	116.8 (3)	O2—C1—C2	127.1 (3)
O1—C5—C4	121.1 (3)	O1—C1—C2	116.9 (3)
C6—C5—C4	122.1 (3)	C9—C8—C7	118.4 (3)
C7—C6—C5	118.1 (3)	C9—C8—H8	120.8
C7—C6—H6	121.0	C7—C8—H8	120.8
C5—C6—H6	121.0	C15—C14—F1	119.6 (4)
C8—C9—C4	122.0 (3)	C15—C14—C13	123.1 (4)
C8—C9—H9	119.0	F1—C14—C13	117.3 (4)
C4—C9—H9	119.0	C3—C2—C1	121.7 (3)
C9—C4—C5	117.4 (3)	C3—C2—H2	119.2
C9—C4—C3	124.9 (3)	C1—C2—H2	119.2
C5—C4—C3	117.8 (3)	C14—C15—C16	118.9 (4)
C16—C11—C12	119.2 (3)	C14—C15—H15	120.6
C16—C11—C10	121.7 (3)	C16—C15—H15	120.6
C12—C11—C10	119.0 (3)	C14—C13—C12	118.0 (4)
C15—C16—C11	120.4 (3)	C14—C13—H13	121.0
C15—C16—H16	119.8	C12—C13—H13	121.0
			
C10—O3—C7—C6	−122.3 (3)	C12—C11—C16—C15	0.4 (5)
C10—O3—C7—C8	63.4 (4)	C10—C11—C16—C15	−178.8 (3)
C7—O3—C10—O4	1.1 (5)	C9—C4—C3—C2	−179.0 (3)
C7—O3—C10—C11	179.3 (3)	C5—C4—C3—C2	1.3 (5)
C1—O1—C5—C6	178.7 (3)	C16—C11—C12—C13	−0.2 (5)
C1—O1—C5—C4	−0.7 (4)	C10—C11—C12—C13	179.0 (3)
C8—C7—C6—C5	1.0 (4)	C5—O1—C1—O2	−177.7 (3)
O3—C7—C6—C5	−173.3 (3)	C5—O1—C1—C2	1.5 (4)
O1—C5—C6—C7	−179.7 (3)	C4—C9—C8—C7	1.4 (5)
C4—C5—C6—C7	−0.3 (4)	C6—C7—C8—C9	−1.5 (5)
C8—C9—C4—C5	−0.8 (5)	O3—C7—C8—C9	172.5 (3)
C8—C9—C4—C3	179.5 (3)	C4—C3—C2—C1	−0.5 (5)
O1—C5—C4—C9	179.6 (3)	O2—C1—C2—C3	178.2 (4)
C6—C5—C4—C9	0.2 (4)	O1—C1—C2—C3	−0.9 (5)
O1—C5—C4—C3	−0.7 (4)	F1—C14—C15—C16	180.0 (3)
C6—C5—C4—C3	179.9 (3)	C13—C14—C15—C16	−0.4 (6)
O4—C10—C11—C16	176.2 (3)	C11—C16—C15—C14	−0.1 (5)
O3—C10—C11—C16	−1.9 (4)	C15—C14—C13—C12	0.6 (6)
O4—C10—C11—C12	−3.0 (5)	F1—C14—C13—C12	−179.7 (3)
O3—C10—C11—C12	178.9 (3)	C11—C12—C13—C14	−0.3 (6)

### Hydrogen-bond geometry (Å, º)

Cg2 and Cg4 are the centroids of the C4–C9 benzene ring and the coumarin ring system, respectively.

**Table:**

*D*—H···*A*	*D*—H	H···*A*	*D*···*A*	*D*—H···*A*
C16—H16···O3	0.93	2.37	2.693 (4)	100
C2—H2···O2^i^	0.93	2.51	3.412 (4)	163
C1—O2···*Cg*2^ii^	1.20 (1)	3.27 (1)	3.403 (3)	86 (1)
C1—O2···*Cg*4^ii^	1.20 (1)	3.57 (1)	3.368 (3)	71 (1)

Symmetry codes: (i) −*x*+2, *y*+1/2, −*z*+2; (ii) *x*−1, *y*, *z*.

### Table S1

Experimental and calculated bond lengths (Å)

**Table:**

Bond	X-ray	6-311^++^G(d,p)
O1—C5	1.374 (3)	1.348
O1—C1	1.385 (4)	1.354
O3—C10	1.350 (4)	1.342
O3—C7	1.398 (4)	1.375
C7—C6	1.374 (4)	1.373
C7—C8	1.387 (5)	1.3889
C10—O4	1.202 (4)	1.180
C10—C11	1.473 (4)	1.486
C5—C6	1.379 (4)	1.385
C5—C4	1.399 (4)	1.385
O2—C1	1.202 (4)	1.178
C9—C8	1.373 (5)	1.374
C9—C4	1.388 (4)	1.395
C4—C3	1.434 (4)	1.452
C11—C16	1.388 (5)	1.390
C11—C12	1.388 (5)	1.391
C16—C15	1.378 (5)	1.383
F1—C14	1.355 (4)	1.321
C3—C2	1.332 (5)	1.329
C12—C13	1.383 (5)	1.380
C1—C2	1.451 (5)	1.468
C14—C15	1.352 (6)	1.378
C14—C13	1.374 (7)	1.379

### Table S2

Experimental and calculated bond angles (°)

**Table:**

Bond angle	X-ray	6-311^++^G(d,p)
C5—O1—C1	121.8 (2)	123.7
C10—O3—C7	120.5 (3)	119.9
C6—C7—C8	122.1 (3)	122.0
C6—C7—O3	115.8 (3)	117.7
C8—C7—O3	121.9 (3)	120.1
O4—C10—O3	122.7 (3)	123.1
O4—C10—C11	126.0 (3)	124.8
O3—C10—C11	111.2 (3)	112.1
O1—C5—C6	116.8 (3)	117.1
O1—C5—C4	121.1 (3)	121.4
C6—C5—C4	122.1 (3)	121.5
C7—C6—C5	118.1 (3)	118.2
C8—C9—C4	122.0 (3)	121.0
C9—C4—C5	117.4 (3)	118.6
C9—C4—C3	124.9 (3)	124.2
C5—C4—C3	117.8 (3)	117.2
C16—C11—C12	119.2 (3)	119.7
C16—C11—C10	121.7 (3)	122.4
C12—C11—C10	119.0 (3)	117.8
C15—C16—C11	120.4 (3)	120.3
C2—C3—C4	120.7 (3)	120.5
C13—C12—C11	120.5 (4)	120.5
O2—C1—O1	116.0 (3)	118.7
O2—C1—C2	127.1 (3)	124.9
O1—C1—C2	116.9 (3)	116.3
C9—C8—C7	118.4 (3)	118.7
C15—C14—F1	119.6 (4)	118.7
C15—C14—C13	123.1 (4)	122.6
F1—C14—C13	117.3 (4)	118.7
C3—C2—C1	121.7 (3)	121.0
C14—C15—C16	118.9 (4)	118.5
C14—C13—C12	118.0 (4)	118.3

### Table S3

Experimental and calculated torsion angles (°)

**Table:**

Torsion angle	X-ray	6-311^++^G(d,p)
C10—O3—C7—C6	-122.3 (3)	-109.7
C10—O3—C7—C8	63.4 (4)	73.7
C7—O3—C10—O4	1.1 (5)	-0.1
C7—O3—C10—C11	179.3 (3)	179.9
C1—O1—C5—C6	178.7 (3)	-180.0
C1—O1—C5—C4	-0.7 (4)	-0.1
C8—C7—C6—C5	1.0 (4)	-0.2
O3—C7—C6—C5	-173.3 (3)	-176.7
O1—C5—C6—C7	-179.7 (3)	179.9
C4—C5—C6—C7	-0.3 (4)	-0.0
C8—C9—C4—C5	-0.8 (5)	0.0
C8—C9—C4—C3	179.5 (3)	-179.9
O1—C5—C4—C9	179.6 (3)	-179.7
C6—C5—C4—C9	0.2 (4)	0.1
O1—C5—C4—C3	-0.7 (4)	0.2
C6—C5—C4—C3	179.9 (3)	-179.9
O4—C10—C11—C16	176.2 (3)	-179.7
O3—C10—C11—C16	-1.9 (4)	0.3
O4—C10—C11—C12	-3.0 (5)	0.4
O3—C10—C11—C12	178.9 (3)	-179.6
C12—C11—C16—C15	0.4 (5)	-0.1
C10—C11—C16—C15	-178.8 (3)	179.9
C9—C4—C3—C2	-179.0 (3)	179.8
C5—C4—C3—C2	1.3 (5)	-0.2
C16—C11—C12—C13	-0.2 (5)	0.0
C10—C11—C12—C13	179.0 (3)	-180.0
C5—O1—C1—O2	-177.7 (3)	180.0
C5—O1—C1—C2	1.5 (4)	-0.1
C4—C9—C8—C7	1.4 (5)	-0.3
C6—C7—C8—C9	-1.5 (5)	0.4
O3—C7—C8—C9	172.5 (3)	176.8
C4—C3—C2—C1	-0.5 (5)	-0.0
O2—C1—C2—C3	178.2 (4)	-179.8
O1—C1—C2—C3	-0.9 (5)	0.1
F1—C14—C15—C16	180.0 (3)	180.0
C13—C14—C15—C16	-0.4 (6)	-0.0
C11—C16—C15—C14	-0.1 (5)	0.1
C15—C14—C13—C12	0.6 (6)	-0.0
F1—C14—C13—C12	-179.7 (3)	180.0
C11—C12—C13—C14	-0.3 (6)	0.0
